# COVID-19 vaccination associated severe immune thrombocytopenia

**DOI:** 10.1186/s40164-021-00235-0

**Published:** 2021-07-15

**Authors:** Syed Raza Ali Shah, Sherpa Dolkar, Jacob Mathew, Prakash Vishnu

**Affiliations:** 1 Departments of Hospital Medicine, St. Michael Medical Center, Virginia Mason Franciscan Health, Silverdale, WA USA; 2 Departments of Hematology, St. Michael Medical Center, Virginia Mason Franciscan Health, Silverdale, WA USA; 3 4655 Olivine Dr SW, Port Orchard, WA 98367 USA

**Keywords:** Immune thrombocytopenia, Coronavirus disease 2019, Pfizer-BioNTech COVID-19 vaccine, Johnson and Johnson COVID-19 vaccine

## Abstract

**Background:**

Coronavirus disease 2019 (COVID-19) caused by severe acute respiratory syndrome coronavirus 2 has emerged as a deadliest global pandemic after its identification in December 2019 in Wuhan, China resulting in more than three million deaths worldwide. Recently FDA issued emergency authorization for three vaccines for prevention of COVID-19. Here in, we report three cases of severe immune thrombocytopenia (ITP) following COVID-19 vaccination and their clinical course.

**Case presentations:**

Case #1: 53 year old male with past medical history of Crohn’s disease was admitted for myalgias and diffuse petechial rash 8 days after receiving second dose of Pfizer-BioNTech COVID-19 vaccine. A complete blood test showed a platelet count of 2 × 10^9^/L. Patient did not have a prior history of thrombocytopenia and other causes of thrombocytopenia were ruled out by history and pertinent lab data. He received two doses of intravenous immunoglobulin and oral dexamethasone for 4 days resulting in normalization of platelet counts.

Case #2: 67 year male with past medical history of chronic ITP in remission was admitted for melena 2 days after receiving his first dose of Pfizer-BioNTech COVID-19 vaccine. A complete blood test showed a platelet count of 2 × 10^9^/L. Physical exam showed generalized petechiae. There was no history of recent flares of ITP and patient had normal platelet counts following his splenectomy 4 years ago. He received two doses of IVIG and oral dexamethasone for 4 days with gradual improvement in platelet counts.

Case #3: 59 year old female with past medical history of chronic ITP secondary to SLE was admitted for bloody diarrhea 2 days after receiving her first dose of Johnson and Johnson COVID-19 vaccine. Physical exam was unremarkable. A complete blood test showed platelet count of 64 × 10^9^/L which dropped to 27 × 10^9^/L during hospital course. She received oral dexamethasone for 4 days with improvement in platelet counts.

**Conclusion:**

COVID-19 vaccination induced ITP has been recently acknowledged. However, given very few cases and limited data, currently there are no guidelines for management of ITP caused by COVID-19 vaccine as well as vaccination of people with predisposing conditions.

## Introduction

Immune thrombocytopenia (ITP) is an acquired hemorrhagic diathesis characterized by a platelet count of < 100 × 10^9^/L caused by immune-mediated destruction of platelets, impaired production or increased splenic sequestration [1]. ITP is idiopathic in 80% of cases, and primary ITP is often thought to be an autoimmune condition. However, 20% of cases of ITP are usually secondary to an underlying precipitating etiology such as infection, medications, rheumatologic disorders or malignancy. Occurrence of ITP has also been reported following vaccinations against various infectious agents especially measles-mumps-rubella (MMR), but also *Hemophilus influenza*, hepatitis B virus, human papilloma virus, varicella-zoster, polio and pneumococcus [2]. Recently, FDA issued emergency use authorization for three vaccines for the prevention of coronavirus disease 2019 (COVID-19) caused by severe acute respiratory syndrome coronavirus 2 (SARS-CoV-2) [3]. Few cases of ITP after SARS-CoV-2 vaccination with both the Pfizer and Moderna vaccines have been reported and have reached public attention. Herein, we report three cases of severe ITP that occurred following COVID-19 vaccination and their outcome.

## Case presentations

### Case #1

**Fig. 1:**
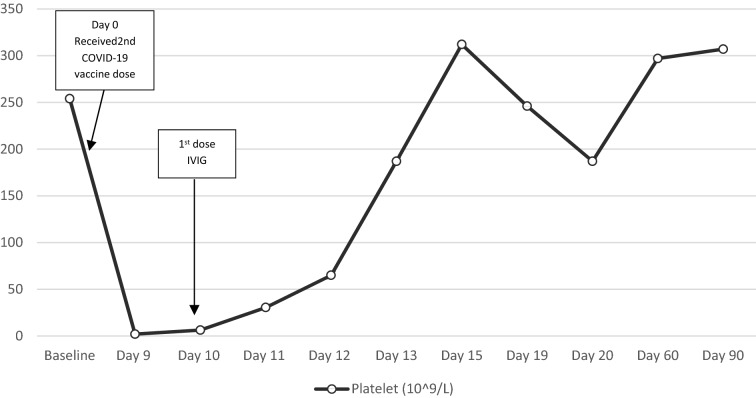
Platelet count before, during and after hospitalization for ITP with timeline of relevant events

A 53-year-old man with past medical history of Crohn’s disease well-controlled with Ustekinumab, and no prior history of thrombocytopenia or exposure to heparin was seen in Emergency Department at St. Michael Medical Center Silverdale, Washington (ED) with symptoms of intermittent episodes of high-grade fever with chills, diffuse myalgia and petechial rash over chest, abdomen and extremities that had started 2 days prior. He did not have symptoms of coryza, cough, headache, abdominal pain, rectal bleeding, dysuria, diarrhea or altered sensorium. He had received the second of the two doses of Pfizer-BioNTech COVID-19 vaccine 8 days prior to the onset of symptoms. In the ED, he had a temperature of 39 °C. He had petechiae in the oropharynx, anterior trunk and bilateral upper and lower extremities. Rest of the physical exam was unremarkable. A complete blood count (CBC) showed a platelet count of 2 × 10^9^/L. Rest of the blood counts and red cell indices were normal. Peripheral blood film did not show platelet clumping or schistocytes. Current list of medications didn’t reveal any culprit medications and he had normal platelet count (254 × 10^9^/L) 5 weeks prior. Urinalysis, Blood cultures and X-ray chest did not show any evidence of infectious etiology and he remained afebrile throughout hospital course. Viral respiratory panel including influenza A and B, RSV and COVID-19 was negative. Hepatitis C and HIV serology was non-reactive. Coagulation profile was normal. Computed tomography (CT) of the head, chest, abdomen and pelvis did not show any evidence of thrombosis. Spleen measured 14.2 cm. Complement levels and autoimmune panel including anti double stranded DNA antibody and anti CCP antibody was normal. Bone marrow biopsy showed normocellular bone marrow with mildly increased megakaryopoiesis, preserved trilineage hematopoiesis without atypia or increased blasts. Flow cytometry didn’t show any abnormal B cells without an increase in CD 34 positive blasts. He received intravenous immunoglobulin (IVIG) 1 g/kg/day for 2 consecutive days and oral dexamethasone 40 mg/day for 4 consecutive days. With this regimen, the platelet count normalized in about 4 days after initiation of therapy and the response was durable. (Fig. 1).

### Case #2

**Fig. 2:**
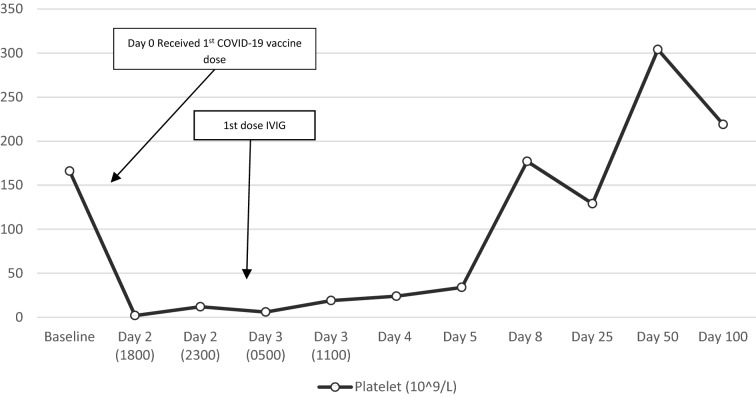
Platelet count before, during and after hospitalization for ITP with timeline of relevant events

A 67-year-old man with past medical history of seizure disorder, atrial fibrillation, chronic ITP in remission and no prior exposure to heparin, was seen in ED with symptoms of generalized weakness and melena. Patient reported that he had received the first dose of Pfizer-BioNTech COVID-19 vaccine two days prior to admission. Patient denied any recent flares of ITP and he had normal platelet counts in the range of 140–160 × 10^9^/L since undergoing splenectomy 4 years ago. Physical exam showed generalized petechiae. Rectal exam revealed black tarry stools. Rest of the physical exam was unremarkable. A CBC showed a platelet count of 2 × 10^9^/L. Rest of the blood counts and red cell indices were normal. Peripheral blood film did not show platelet clumping or schistocytes. Hepatitis C and HIV serology was non-reactive. Viral respiratory panel including influenza A and B, RSV and COVID-19 was negative. Coagulation profile was normal. Patient received two units of single-donor platelet transfusion without any significant improvement in platelet count, however melena stopped. CT of the head and chest were unremarkable while the CT of the abdomen showed mild rectal wall thickening with fat stranding suspicious of proctitis. The patient received IVIG 1 g/kg/day for 2 consecutive days and oral dexamethasone 40 mg/day for 4 consecutive days. He had a gradual improvement in platelet count which eventually normalized in about a week after initiation of therapy. (Figure 2) Patient was advised to defer the second dose of COVID-19 vaccine due to the occurrence of severe thrombocytopenia.

### Case #3

**Fig. 3:**
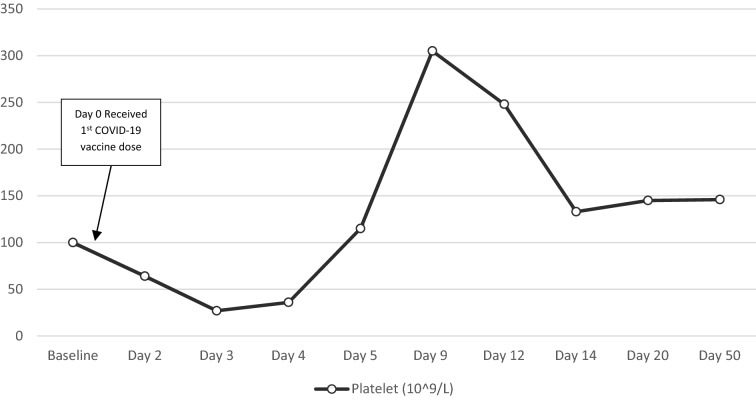
Platelet count before, during and after hospitalization for ITP with timeline of relevant events

A 59-year-old woman with past medical history of chronic ITP and systemic lupus erythematosus (SLE) on hydroxychloroquine was seen in ED for symptoms of abdominal cramps and diarrhea that started 2 days after she had received Johnson and Johnson Covid-19 vaccine. Patient’s previous flare of ITP was two years ago which was precipitated by Shingrex vaccine that was treated successfully with steroids, following which her platelet count remained stable (~ 100 × 10^**9**^/L). Physical exam was unremarkable. A complete blood test showed a platelet count of 64 × 10^**9**^/L and subsequently dropping to 27 × 10^9^/L the following day. Peripheral blood film did not show platelet clumping or schistocytes. CT abdomen showed mild distal colitis. Prior laboratory data showed non-reactive HIV and hepatitis B and C serology. Patient received oral dexamethasone 40 mg/day for 4 days with gradual improvement in platelet count as shown in Fig. 3.

## Discussion

Vaccination associated ITP is a rare phenomenon. ITP after COVID19 vaccination is relatively new occurrence, and the management and outcomes of these cases are not well published in medical literature. Only recently, a case series reported twenty patients with thrombocytopenia following vaccination, based on the data obtained from the Centers for Disease Control and Prevention (CDC), FDA and agencies of the U.S. Department of Health and Human Services (HHS) Vaccine Adverse Events Reporting System (VAERS) [4]. Also, an anecdotal case of a 56-year-old physician who had developed severe thrombocytopenia after receiving COVID-19 vaccine and eventually died due to hemorrhagic stroke was reported in The New York Times [5].

Our report describes three cases of severe ITP that occurred following COVID-19 vaccination and also provides insights into the treatment and outcomes for ITP secondary to COVID-19 vaccination. All three patients responded well to treatment with corticosteroids ± IVIG. If the treatments (IVIG and/or steroids) utilized in our cases were not effective, and the platelet count remained very low, it would then be reasonable to escalate therapy to include a thrombopoietic agent, and potentially vinca alkaloids, depending upon response. Avoiding rituximab from initial treatment would be appropriate since the response to rituximab may take up to 6–8 weeks; furthermore, the response to COVID-19 vaccine may be impaired by rituximab. Whether post COVID-19 vaccination ITP cases will prove to be self-limiting or persist and lead to chronic ITP remains uncertain. It is also unclear if the ITP is secondary to the vaccination or primary ITP that was coincident with the vaccine. Nevertheless, the clinical manifestation and the positive response to treatment akin to ITP, such as with corticosteroids ± IVIG, suggest an antibody‐mediated platelet destruction.

ITP has been previously reported with different types of vaccines including hepatitis B virus (HBV) human papilloma virus (HPV) varicella zoster, haemophilus influenza, polio, pneumococcus, Diphtheria-tetanus-acellular-pertussis (DTap) and especially measles-mumps rubella (MMR) [6]. Proposed mechanisms include molecular mimicry and antibody generation to adjuvants/additives included in the vaccine composition which can lead to generation of autoantibodies that cause platelet destruction and inhibit platelet production [7]. Data also show that post-vaccination ITP ranges in severity from mild to moderate to severe and is highly responsive to IVIG treatment as seen in our patients [8]. Literature review shows conflicting data as some studies show evidence of ITP reactivation when exposed to the same vaccine while others do not demonstrate the ITP reactivation when the vaccine is repeated [9–12]. Many authors believe ITP is not an absolute contraindication for vaccination given a much higher rate of ITP linked with natural infection as compared to vaccination however recommend to avoid vaccination during active ITP flare [13].

## Conclusion

As mass vaccination is in progress, more data should be collected to establish a cause-effect relationship of COVID-19 vaccination and associated ITP so that the CDC can formulate guidelines for safe administration for COVID-19 vaccination. This can be done by outlining possible contraindications and potential treatment measures for ITP so as to prevent fatal outcomes. Additionally, more research may be needed to make changes in vaccine composition to prevent thrombocytopenia. One consideration could be to monitor patients with history of ITP for an antibody response and reserving the second dose of the vaccine for those without an adequate response. Until there is further development of appropriate guidelines, physicians should discuss these risks and benefits.

## Data Availability

The data used during the current study is available from the corresponding author on reasonable request.

## Abbreviations

ITP: Immune thrombocytopenia
COVID-19: Coronavirus disease 2019
SARS-CoV-2: Severe acute respiratory syndrome coronavirus 2
ED: Emergency Department
CBC: Complete blood count
CT: Computed tomography
IVIG: Intravenous immunoglobulin
SLE: Systemic lupus erythematosus
CDC: Centers for Disease Control and Prevention
FDA: U. S. Food and Drug Administration
HHS: Department of Health and Human Services
VAERS: Vaccine adverse events reporting system
HBV: Hepatitis B virus
HPV: Human papilloma virus
DTap: Diphtheria-tetanus-acellular-pertussis
MMR: Measles-mumps rubella
